# Sustainable, healthy cities: making the most of the urban transition

**DOI:** 10.1186/s40985-016-0037-0

**Published:** 2016-10-28

**Authors:** José Gabriel Siri

**Affiliations:** United Nations University International Institute for Global Health (UNU-IIGH), Federal Territory of Kuala Lumpur, Malaysia

**Keywords:** Global environmental change, Healthy cities, Systems thinking, Urban policy, Sustainability, Urban transition, Urban health, Planetary health

## Abstract

The world is undergoing a massive urban transition, which is now both the greatest driver of global environmental change and the most significant influence on human health. Cities offer real opportunities for improving health, but managed poorly, they can also create or reinforce significant health deficits while putting severe stresses on the natural systems which support human civilization. Management of urban problems is rarely straightforward, as complexity across scales and sectors, in causal structures, actors and incentives, can lead to ineffective policies and unintended consequences. Systems thinking offers a promising way forward in its ability to deal with non-linear relationships and simultaneous actions and outcomes. Encompassing, on the one hand, analytic frameworks and methods that can provide important causal insights and a test bed for urban policy, and on the other, broad processes of inter- and trans-disciplinary engagement to better define problems and feasible solutions, systems approaches are critical to the current and future design and management of sustainable healthy cities.

## Main text

The current moment of urban transition offers a unique opportunity to consider how to live healthier lives on a healthier planet. Cities, and the process of urbanization, are critical moderators of the interplay between human health and sustainability.

It is clear that the urban touches on nearly every aspect of environmental change and virtually every facet of the modern human condition. It is only in the last few generations that substantial fractions of the human race began living in cities, yet we are now in the middle of an unprecedented urban transition. Although the timing of this transition has varied from place to place, the great-grandparents of the vast majority of people alive today were born in rural areas, as has been the norm throughout history. Yet, the last century has seen a dramatic shift of people to cities, and during the last decade we passed the point where most human beings are urban beings [1]. Based on the expected growth of urban populations through the middle of the twenty-first century, close to 0.2 million people will move to or be born in cities around the world today, like any other day. By 2050, 2.4 billion people will be added to the global urban population and two thirds of all humans will live in cities [2]. This transition is economic, social, cultural, and, perhaps most of all, ecological. It may indeed be the most radical shift in habitat the human species has ever experienced.

The urban transition manifests not only in people but also in space, on a scale which is perhaps less easy to appreciate. It has been estimated that global urban extents will increase by 185 % in the interval between 2000 and 2030 [3]. That is to say, in one generation, humanity will nearly triple the urban area that accumulated over 7000 years of civilizational growth. In parts of the world, we have already experienced such transitions. To compare the lives of the residents of Shanghai or Dubai or Kuala Lumpur today to those of a quarter-century ago is to acknowledge vast differences in sensory, aesthetic, physical, and cognitive experiences, not to mention influences on health. The urbanization of the generation to come will echo this vivid transformation but will also be strikingly novel, as people interact with new technologies and social movements and ideas, and increasingly with the consequences of global environmental change.

It is evident that the urban transition coincides with massive shifts in the planetary systems which support human life [4]. Warning signs are clear and wide-ranging, from rising temperatures and sea level to ocean acidification, loss of forests and biodiversity and increased impacts from extreme events [4]. And yet, we also see strong net improvements in the human condition: less poverty, longer lives, better health, and more opportunity. The concurrence of these varied trends is no fluke. Cities cover just 3 % of the land surface of the Earth but are responsible for as much as 80 % of all greenhouse gas emissions, three quarters of natural resource consumption, and half of all waste [5]. And yet, cities also generate 80 % or more of global economic output, providing livelihoods and opportunities and offering many the chance to lift themselves out of poverty—though they also exacerbate poverty in some circumstances. Cities act to concentrate the vast majority of human innovation, including in medical, green and other technologies, art and architecture, culture, politics and science—indeed, over 90 % of global patents originate in metropolitan urban settings representing just 23 % of the global urban population [6]. Essentially, cities have come to drive the world, in all its goods and bads.

The urban push has brought improved health, and city dwellers tend to be healthier than others, and yet a broad range of urban health challenges remain or are emerging. This includes a rising tide of non-communicable diseases [7], mental health problems [8], issues related to exposure to growing climate risks [9], drug-resistant pathogens, [10] substance abuse [11], crime [12], and others. Table 1 briefly reviews some of these challenges, for many of which the particular role of the urban environment remains incompletely understood. Just as is the case for wealth, there is also a great deal of inequity in the distribution of good health. In some cases, poor, informal or transitional urban neighbourhoods experience health outcomes worse than those observed in rural areas [13]. Often, urban ill health reflects a mismatch between the forms and functions of cities and the evolved needs of the human species. Cities are designed for narrow economic goals or technical efficiencies—for automobiles, in some cases, rather than for people. In other cases, these persistent problems are the result of unrecognized or unaddressed complexity.

**Table 1:** Selected urban health challenges

Health challenge	Description
Non-communicable diseases (NCDs)	Where urban form is associated with sedentary lifestyles or increased consumption of unhealthy foods, city dwellers are at higher risk for obesity and associated NCDs, including heart disease and stroke, hypertension, diabetes and some cancers. Urban air pollution can impair lung function and give rise to or exacerbate cardiovascular and respiratory diseases, including asthma, in acute and chronic forms. It can also contribute to the development of allergic disease or cancer and cause adverse pregnancy outcomes.
Infectious diseases	The high concentration of humans in cities naturally promotes increased transmission of some infectious diseases, such as tuberculosis. Growing mobility within and between cities and urban expansion into natural habitats contribute to ever-more-rapid emergence and spread of infections.
Accidents and injuries	City forms that encourage automobile use, often in combination with inadequate safety regulation, contribute to higher rates of road traffic accidents and deaths.
Mental health	The urban built environment can adversely affect mental health. For example, lack of public space for recreation and socialization can lead to isolation and depression. Noise pollution and commuting can create significant stress.
Disaster risk	Cities are often built in high-risk areas, such as along coastlines and rivers or on hill slopes. In particular, poor informal settlements often take hold and expand on otherwise unwanted land at high risk, such as on floodplains. Urban expansion can compromise fertile agricultural land, decreasing food security.
Climate change	Long-term urban impacts on climate can harm health directly (e.g. through increases in acute heat waves or other extreme climatic events) or indirectly (e.g. through shifts in vector ranges or resource availability).

One characteristic of many of these interlinked challenges is their intractability. A recent study found that, of 180 countries examined, not one had reduced its rates of obesity and overweight in the preceding three decades [14]. This belies a mass of research on the causes and control of obesity and overwhelming incentive—obesity costs over US$2 trillion per year globally [15]. Why should this challenge be so difficult to address? One problem is that scientific analysis and policy action tend to be reductive. They often aim to identify causal effects between individual inputs and outcomes, controlling for all other factors, and then to act on this narrow understanding. Yet, reality is not simple—in fact, it is quite slippery. In the case of obesity, many different mechanisms entangle in complex feedback loops to generate the observed outcomes [16]. Each variable may imply many different potential interventions, while convoluted pathways and multiple connections often lead to unintended consequences. Within this framework, simultaneous decisions are made by actors at all scales. Moreover, the causal system that generates obesity and overweight is just one of numerous sets of systems of interest in the urban context. Traditional approaches tend to perform less than optimally in the face of such complexity [17].

Fortunately, there are ways forward. Systems thinking is increasingly recognized as a framework that allows us to address complex issues of urban health and sustainability [18–20]. In particular, this involves analytical methods that can handle feedbacks and complex non-linear relationships among variables as well as broad processes of engagement among different disciplines and between researchers, decision makers, individuals, and communities. It has become apparent that health in cities is deeply connected and interlinked with sustainability. Systems thinking offers a way to attack intractable health issues while also addressing a set of deeper challenges related to the speed and scale of growth, equity in a world of scarce resources, sustainability, resilience, and governance. Recognition of the integrated nature of such challenges has already led, in some contexts, to the identification and application of actions that produce co-benefits for a wide range of urban health and sustainability challenges, such as promoting active public transport over private car use.

The year 2015 was momentous, highlighted by major agreements on disaster risk [21], sustainable development [22], development financing [23], and climate change [24]. This process was marked by a more direct recognition of the crucial role that cities play in human affairs than ever before—this is particularly evident in the adoption of Sustainable Development Goal 11 on sustainable cities and communities. At the same time, funders like the Rockefeller Foundation and the Wellcome Trust have become ever-more conscious of the need for systems thinking, which is inherent in the idea of planetary health. Research efforts like the new International Council for Science programmes on Systems Thinking for Urban Health [25] and Future Earth [26] are also embracing this paradigm, as are global research institutes like the United Nations University’s International Institute for Global Health [27], and scores of other academic institutions around the world. It is to be hoped that this collective effort will lead to a strong place for systems thinking about health and sustainability in the implementation of the New Urban Agenda that has emerged from Habitat III, the United Nations Conference on Housing and Sustainable Urban Development,  in late 2016 [28]. In the meantime, we must take hold of the unique opportunity represented by the urban transition to push for a sustainable, healthy planet, full of sustainable, healthy cities.
